# Atypical MEGDHEL Syndrome: A Milder Phenotype With Hepatic Presentation and Failure to Thrive Associated With a Homozygous Nonsense Variant of *SERAC1*

**DOI:** 10.1002/jmd2.70017

**Published:** 2025-05-12

**Authors:** Rita Marchante Pita, Raquel Amaral, Laura Vilarinho, Luísa Diogo, Isabel Gonçalves, Susana Nobre

**Affiliations:** ^1^ Department of Pediatrics, Unidade Local de Saúde de Coimbra Portugal; ^2^ Department of Pediatrics Hospital Divino Espírito Santo, Ponta Delgada Azores Portugal; ^3^ Department of Human Genetics National Institute of Health Dr Ricardo Jorge Porto Portugal; ^4^ Reference Center of Hereditary Metabolic Diseases, Unidade Local de Saúde de Coimbra Coimbra Portugal; ^5^ Faculty of Medicine of University of Coimbra Coimbra Portugal

**Keywords:** 3‐methylglutaconic aciduria, hepatopathy, MEGDEL syndrome, MEGDHEL syndrome, pediatrics, *SERAC1* gene

## Abstract

MEGDHEL syndrome, caused by a *SERAC1* gene defect, is clinically defined as the association of 3‐MGA‐uria (MEG), deafness (D), hepatopathy (H), encephalopathy (E), and Leigh‐like features (L). Clinical presentation typically begins in the neonatal period, with neurological symptoms becoming more evident by 2 years of age. Severe liver involvement has also been reported. We report the case of a 3‐year‐old boy with increased transaminases and failure to thrive of unknown cause. He was born prematurely at 35 weeks and needed neonatal intensive care support for 24 h due to transient tachypnea. At 18 months, laboratory investigations for failure to thrive revealed elevated transaminases without cholestasis, which persisted on subsequent evaluations. Abdominal wall collateral veins were found during physical examination, and the liver ultrasound revealed steatosis, prompting the decision to proceed with a liver biopsy. Common causes of chronic liver disease were ruled out. Following liver biopsy, performed under general anesthesia, he had an episode of unexplained decompensation (metabolic acidosis, hyperlactatemia, and 3‐methylglutaconic aciduria). The aciduria persisted upon subsequent evaluation. Liver histology showed macro/microvesicular steatosis (25%), portal tract inflammation, and mild fibrosis. Cardiac evaluation, along with brain magnetic resonance imaging and spectroscopy, was normal. Further investigations revealed decreased hepatic activity of respiratory mitochondrial chain complexes and marginal mtDNA depletion (28.1%). Analysis of the *SERAC1* gene showed homozygosity for p.Y259* (c.777T>G, exon 9). This case report raises awareness for an atypical presentation of MEGDHEL syndrome associated with a homozygous nonsense variant of SERAC1 clinically characterized by mild hypertransaminasemia, failure to thrive, no neurological involvement, and starting in early childhood rather than infancy.

1

**Summary**

- MEGDHEL syndrome is a rare autosomal recessive disorder due to variants in the SERAC1 gene.
- MEGDHEL syndrome is characterized by 3‐methylglutaconic aciduria (MEG), deafness (D), hepatopathy (H), encephalopathy (E) and Leigh‐like features (L).
- We report an atypical presentation of MEGDHEL syndrome characterized by mild hepatopathy and failure to thrive, without neurological involvement, associated with a homozygous nonsense SERAC1 variant, expanding the clinical spectrum of the syndrome.

## 2 Background

The 3‐methylglutaconic aciduria (3‐MGA‐uria) syndromes comprise a heterogeneous group of inborn errors of metabolism characterized by increased urinary excretion of 3‐methylglutaconic acid and distinct molecular and clinical presentation [1, 2]. In 2012, Wortmann et al. proposed a classification for inborn errors of metabolism with 3‐MGA‐uria based on the underlying pathomechanism [2]. This classification includes the “primary 3‐MGA‐uria” or 3‐methylglutaconyl‐CoA hydratase deficiency (former type I), caused by the hydratase defect in leucine catabolism (AUH defect; OMIM #250950) and the “secondary 3‐MGA‐urias” (former type II to V), where the mechanism leading to the 3‐MGA‐uria is unknown but not directly related to the leucine breakdown. The “secondary 3‐MGA‐urias” can be divided into two distinct groups: (1) defective phospholipid remodeling (*TAZ* defect/Barth syndrome [OMIM #302060]; *SERAC1* defect/MEGDHEL syndrome [OMIM #614739]); (2) mitochondrial membrane‐associated disorders (*OPA3* defect/Costeff syndrome [OMIM #258501]; *DNAJC19* defect/DCMA syndrome [OMIM #610198]; *TMEM70* defect [OMIM #614052]) [2].

MEGDEL syndrome, a “secondary 3‐MGA‐uria” (former type IV), was first described in 2006 by Wortmann et al. It is a rare mitochondrial disorder with autosomal recessive inheritance that is clinically defined as the association of 3‐MGA‐uria (MEG), deafness (D), encephalopathy (E), and Leigh‐like features (L) [3, 4, 5]. When patients also present with hepatopathy (H), the syndrome is referred to as MEGDHEL [3]. In 2012, the MEGDHEL syndrome was associated to mutations in the *SERAC1* (serine active site‐containing 1) gene [6]. This gene encodes a serine‐lipase domain containing protein, with phosphatidylglycerol remodeling activity, located at the contact site between mitochondria and endoplasmic reticulum, that is essential for mitochondrial function and intracellular cholesterol trafficking [6, 7]. More recently, Fang et al. [8], suggested that SERAC1 may be part of the mitochondrial serine import system. The authors found that the loss of SERAC1 in mice, HEK293T cells, and patient‐derived cells impaired the function of the mitochondrial serine importer sideroflexin‐1 (SFXN1), disrupting the one‐carbon‐cycle and leading to insufficient supply of nucleotides and depletion of primary mitochondrial DNA (mtDNA).

The clinical presentation of MEGDHEL syndrome typically begins in the neonatal period (infantile‐onset type) with sepsis‐like symptoms and hypoglycemia. During the first year of life, affected infants experience feeding difficulties, failure to thrive, and hypotonia. By the age of 2 years, neurologic involvement becomes apparent, characterized by progressive deafness, psychomotor development delay, dystonia, and spasticity. Affected children may also lose previously acquired skills, making them adult‐dependent for daily activities [9, 10].

Cases of MEGDHEL syndrome starting between the age of 2 and 7 years with a milder and oligosymptomatic phenotype (juvenile‐onset type) or in adulthood with generalized dystonia, have also been reported [11, 12].

We report an atypically mild phenotype of MEGDHEL syndrome, beginning in early childhood and characterized by failure to thrive, mild hypertransaminasemia, and 3‐MGA‐uria, without neurologic involvement, associated with a novel variant in the *SERAC1* gene.

## 3 Case Report

We present a 3‐year‐old boy who was admitted to our center for liver biopsy due to elevated transaminases and failure to thrive of unknown etiology.

He is the first child of healthy, nonconsanguineous parents, born via eutocic delivery at 35 weeks of gestation (precipitous labor), after an uneventful pregnancy. His birth weight was 2070 g (11th percentile) and his Apgar scores at 1, 5, and 10 min were 6, 8, and 9, respectively. One hour after birth, he began to exhibit signs of respiratory distress and was admitted to the neonatal intensive care unit for 24 h due to transient tachypnea. No other significant issues were noted during the neonatal period, including neonatal sepsis, hypoglycemia, liver dysfunction, or hyperbilirubinemia.

During the first 9 months of his life, his growth was normal. However, from that point onward, he showed failure to thrive, crossing from the 25th percentile to below the 3rd percentile. Laboratory investigation revealed increased transaminases (aspartate aminotransferase [AST] 125 IU/L, alanine aminotransferase [ALT] 155 IU/L; reference values < 40 IU/L), normal gamma‐glutamyl transferase (GGT) level (55 IU/L, reference value 12–58 IU/L) and no evidence of cholestasis, which persisted in subsequent evaluations. Liver ultrasound showed steatosis without other structural abnormalities. Total bile acids, ammonia, total protein, and albumin, total cholesterol, prothrombin time, fibrinogen, immunoglobulin G, and alpha‐1 antitrypsin levels were all within age‐appropriate reference values. Plasma amino acids and acylcarnitines were also normal. The family history was negative for liver diseases or inborn errors of metabolism.

At the age of 3 years, during his first evaluation at our center, the physical examination only revealed abdominal wall collateral veins (Grade I). He subsequently underwent a liver biopsy, performed under general anesthesia and without complications. Immediately following the biopsy, he experienced an episode of acute decompensation, characterized by excessive drowsiness and vomiting. Blood tests revealed metabolic acidosis (venous blood gas analysis: pH 7.28, pCO_2_ 43 mmHg, HCO_3_ 20.2 mmol/L, and base excess −6.5 mmol/L), and hyperlactatemia (5.7 mmol/L; reference value 0.4–2.0 mmol/L), without a worsening of liver enzymes or impaired liver function. Urine organic acids analysis showed 3‐MGA‐uria (203 μmol/mmol creatinine; reference value 0–19.0 μmol/mmol creatinine), which persisted in a subsequent evaluation 1 year later. He also underwent a multidisciplinary evaluation with cardiac (electrocardiography and cardiac ultrasound), otorhinolaryngology, ophthalmologic, neurologic (including brain magnetic resonance imaging [MRI] and spectroscopy) and neurocognitive evaluation, all of which were normal.

**FIGURE 1:**
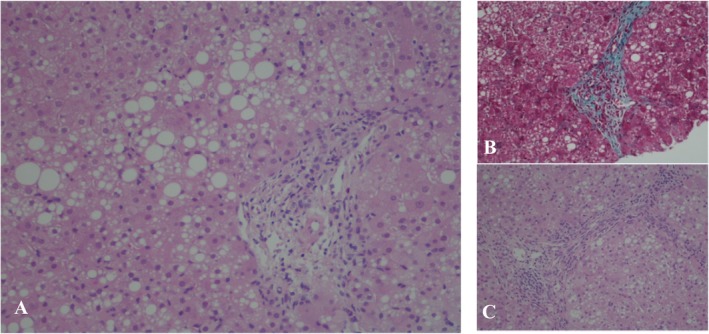
Liver histology images. (A) Macro and microvesicular steatosis and portal fibrosis (×200); (B) Portal fibrosis (×200); (C) Portal tract with fibrotic septae (×200).

Liver histology revealed macro/microvesicular steatosis (25%), portal tract inflammation, and mild fibrosis (Figure 1). Further analysis of the liver tissue showed decreased hepatic activity of respiratory mitochondrial chain complexes I (35.2%), III (24.5%), IV (31.2%), V (27.8%), I + III (33.9%), and II + III (12.2%), for a citrate synthase activity of 122.1 nmol/min/mg of protein (reference value of 99.5 ± 33.5 nmol/min/mg of protein), and mtDNA depletion (28.1%). The activity of respiratory mitochondrial chain complexes was measured and classified according to Grazina methodology [13]. The relative mtDNA copy number was quantified using real‐time PCR, with mtDNA depletion defined as a copy number below 30% of the mean reference value in the most affected tissue.

Due to the suspicion of MEGDHEL syndrome, molecular analysis of the *SERAC1* gene was performed, revealing homozygosity for the p.Y259* (c.777T>G, exon 9) variant. This variant, previously described in heterozygosity but not in homozygosity, introduces a premature termination codon, which we predict to be a pathogenic variant. Both parents were also tested and found not to be carriers of the variant.

Since the diagnosis, he has not required any type of treatment or support. His transaminase levels have spontaneously decreased to the normal range and liver ultrasounds showed no progression of liver disease. He is currently 13 years old, with normal growth and appropriate cognition for his age, attending regular education without learning difficulties or grade retention. He maintains no signs of neurological involvement and the MRI has not been repeated. No hearing loss has been reported.

## 4 Discussion

In 2006, Wortmann et al. reported four patients with a distinct clinical phenotype called MEGDEL association [3]. These patients presented with neuroradiological evidence of Leigh syndrome, sensorineural hearing loss, recurrent lactic acidemia, hypoglycemia, moderately increased 3‐MGA urine excretion, and mitochondrial complex I deficiency. Later, MEGDEL syndrome was associated with *SERAC1* pathogenic mutations [6].

Liver involvement is not always present in MEGDEL syndrome and, when it occurs, it is typically transient [9]. In the neonatal period, liver involvement can range from hepatitis of unknown cause (up to 52%) to prolonged hyperbilirubinemia and, more rarely, liver failure (up to 30%), always in association with the other major MEGDEL features [9]. For children without hepatic involvement during the neonatal period, similar manifestations may develop later in life [9].

Unlike other cases reported in the literature, the liver was the main organ affected in our patient, and all tests ruled out neurological involvement and hearing loss.

In 2013, Sarig et al. [14], proposed renaming the condition to MEGDHEL syndrome, emphasizing infantile hepatopathy as a cardinal feature of this association. They described four children with neonatal hypotonia and liver dysfunction including symptomatic hypoglycemia, lactic acidosis, elevated serum transaminases and GGT levels, coagulopathy, hyperammonemia, and markedly elevated serum α‐fetoprotein. The subsequent development of additional features and the identification of *SERAC1* mutations led to the diagnosis of MEGDHEL association. Recent reports have described cases of MEGDHEL syndrome with infantile‐onset hepatopathy, all exhibiting severe liver involvement [15, 16, 17, 18]. However, no cases of liver disease have been documented in milder juvenile‐onset or adult‐onset phenotypes.

Progressive neurological dysfunction with a characteristic neuroradiological pattern is a major feature of MEGDHEL syndrome. Wortmann et al. [10], studied 30 patients with confirmed SERAC1 mutations, ranging in age from 4 days to 8.1 years at the time of the first MRI and 0.4–14.3 years at follow‐up. All patients showed a distinct, progressive brain MRI pattern including T2 signal changes in the pallidum, swelling of the putamen and caudate nucleus, and a progressive putaminal “eye” sign, which is pathognomonic for MEGDHEL. In contrast, the brain MRI of our patient, performed at the age of 3 years, was normal, while most patients at this age show end‐stage basal ganglia disease after delayed development and dystonia onset between 12 and 18 months. At 13 years of age, our patient remains free of clinical neurological symptoms.

The diagnosis of MEGDHEL syndrome is challenging, particularly in the absence of classic clinical features. The episode of severe prostration following general anesthesia, along with metabolic acidosis and increased urinary 3‐MGA excretion, was an important clinical and biochemical finding that prompted further investigations into a potential inborn error of metabolism.

Several inborn errors of metabolism, including mitochondrial disorders, are associated with increased urinary excretion of 3‐MGA [2]. Healthy individuals excrete small amounts of 3‐MGA in urine (< 20 mmol/mol creatinine), whereas in patients with MEGDHEL syndrome, urinary 3‐MGA excretion generally ranges from 16 to 196 mmol/mol creatinine [1, 2, 4]. The levels of 3‐MGA excretion can fluctuate, making it intermittently undetectable, even in those with clinically and molecularly confirmed diagnoses [2]. Therefore, repeating the urine organic acid analysis or conducting it during metabolic decompensation can be particularly useful in diagnosing secondary 3‐MGA‐uria, including MEGDHEL syndrome.

Our patient, now 13 years old, had an atypical presentation of MEGDHEL syndrome, with mild and transient hepatopathy without neurological involvement to date. However, considering the late‐onset cases reported in the literature [4, 11], longer follow‐up is recommended, particularly to monitor for persistent liver fibrosis despite normal liver function tests.

There is no clear correlation between the type of *SERAC1* variant, the level of 3‐MGA‐uria, clinical phenotypes, and outcomes [9]. Our patient carries a SERAC1 variant, previously described only in heterozygosity, now identified in homozygosity. In our patient, this variant is associated with a milder phenotype starting in early childhood and contributes to expanding the clinical spectrum of SERAC1 deficiency.

To the best of our knowledge, no other cases with a similar presentation have been reported. This phenotype with mild and non‐neurological manifestations may contribute to underdiagnosis and underreporting of similar cases of MEGDHEL syndrome.

## Consent

Informed consent was obtained from the legal guardian for the publication of anonymized data. No identifying patient information is presented in the manuscript.

## Conflicts of Interest

The authors declare no conflicts of interest or financial disclosures.

## Data Availability

The data used in this study are available from the corresponding author upon reasonable request.
